# Virtual Patient-PCP-Hospitalist Care Transition Meeting Before Hospital Discharge

**DOI:** 10.1001/jamanetworkopen.2025.15848

**Published:** 2025-06-13

**Authors:** Jing Li, Matthew D. Reuter, Jennifer M. Schmidt, Nathan Moore, Nickole Forget, Jennifer Carron, Janice K. Ernest, Mark V. Williams

**Affiliations:** 1 Department of Medicine, Division of General Internal Medicine and Population Science, University of Alabama at Birmingham, Birmingham; 2 BJC Medical Group, BJC Health System, St Louis, Missouri; 3 Department of Medicine, Washington University in St Louis, St Louis, Missouri; 4 Center for Clinical Excellence, BJC Health System, St Louis, Missouri

## Abstract

**Importance:**

The fragmented health care system presents significant challenges to promoting trust with hospitalized patients.

**Objective:**

To test the feasibility and perceptions of a digital connection tool for communication among patients, hospitalists, and primary care practitioners (PCPs).

**Design, Setting, and Participants:**

This quality improvement study was conducted as a feasibility pilot study from September 2022 to June 2023 on medical floors across 3 hospitals. Participants included adult inpatients, the hospitalists who managed their care, and the patients’ PCPs. Data were analyzed from September 2023 to April 2025.

**Exposure:**

Structured, video-mediated interaction among the patient, family caregiver (if available), hospitalist, and PCP, conducted before discharge.

**Main Outcomes and Measures:**

Outcomes of interest were patient perceptions, assessed using the Consultation and Relational Empathy (CARE) measure. Open-ended question sought patients’ feedback. Hospitalists’ and PCPs’ perceptions were evaluated using Acceptability of Intervention Measure (AIM), Intervention Appropriateness Measure (IAM), and Feasibility of Intervention Measure (FIM) surveys.

**Results:**

The study sample included 106 participants (mean [SD] age, 63.2 [14.9] years; 55 [51.9%] male). Bedside video meetings were a mean (SD) of 8 (2.8) minutes. Overall score for CARE questions showed a significant improvement, increasing from 40.9 (95% CI, 39.5-42.2) to 47.5 (95% CI, 46.5-48.5) (*P* < .001). Of clinicians, 50 of 95 PCPs (52.6%) and 42 of 56 hospitalists (75.0%) completed surveys. PCPs reported more positive perceptions than hospitalists regarding acceptability (4.1 [95% CI, 3.90-4.38] vs 3.6 [95% CI, 3.34-3.84]; *P* = .006) and appropriateness (4.1 [95% CI, 3.86-4.35] vs 3.7 [95% CI, 3.41-3.94]; *P* = .03). Qualitative feedback highlighted improved information sharing, communication, and collaborative discharge planning. Patients felt more cared for and were more confident about follow-up steps.

**Conclusions and Relevance:**

Findings of this quality improvement study highlighted the value of patient-hospitalist-PCP virtual meetings in addressing gaps in information exchange, increasing patient confidence, and improving clinician perceptions of care coordination. These results supported the feasibility and potential of incorporating such interventions as part of routine discharge procedures. The Centers for Medicare & Medicaid Services could consider incorporating a direct interaction component with hospitalists, patients, and PCPs before hospital discharge into the requirements for Transitional Care Management billing or allow current billing codes to be applied in the hospital setting.

## Key Points

**Question:**

What are patients’, hospitalists’, and primary care practitioners’ perceptions of care and care coordination after a video meeting during hospital discharge?

**Findings:**

In this quality improvement study involving 106 patients, video-mediated discharge meetings were associated with significantly improved patient perceptions of care, and clinicians viewed the intervention as acceptable, appropriate, and feasible, with primary care practitioners rating it more positively than hospitalists. Qualitative feedback highlighted enhanced communication, collaborative discharge planning, and increased patient confidence.

**Meaning:**

These findings suggest that structured video-mediated discharge meetings are a feasible and effective strategy to improve care transitions, patient confidence, and clinician collaboration, warranting consideration for integration into routine discharge practices and inclusion in Centers for Medicare & Medicaid Services Transitional Care Management billing codes.

## Teaser

This quality improvement study examines the feasibility and participant perceptions of a structured, video-mediated interaction among the patient, hospitalist, and primary care practitioner at hospital discharge.

## Introduction

Today’s health care system is a fragmented and complex network involving multiple entities in patient care, creating significant challenges during transitions from hospital to home or postacute care facilities. Patients often feel lost and uncertain about who is responsible for their care^1,2,3,4^ due to communication breakdowns and lack of coordination. This can lead to errors, redundant tests, and inconsistent treatment plans.^4,5,6^

Patients and family caregivers tend to place greater trust in long-standing practitioners, such as primary care practitioners (PCPs).^4^ In contrast, hospitalized patients often feel unknown by the hospitalist overseeing their care. However, direct communication and collaboration among patients, family caregivers, hospitalists, and PCPs rarely occur during this high-risk transition phase. Recent research documents the poor quality of communication and education undertaken at hospital discharge.^7^ When patients perceive their care as uncoordinated or believe health care practitioners are not communicating effectively, trust is eroded.^4^ Literature shows that patients who feel known by practitioners report higher levels of trust and better adherence to care plans,^8,9,10,11^ with trust consistently linked to improved health outcomes^11,12,13,14,15^ and patient satisfaction.^13,16,17,18^

Digital tools that allow hospitalists to communicate virtually with PCPs before hospital discharge, especially while including patients and family caregivers, present a valuable opportunity to build trust and improve care transition. The Care Transitions (CT) Technology to Reinforce Understanding and Support Trust (TRUST) pilot aimed to engage hospitalists and PCPs, test the feasibility of video connection, and assess patient and clinician perceptions.

## Methods

### Design and Setting

This quality improvement study was approved by the Washington University Institutional Review Board. All patients provided written informed consent, and PCPs and hospitalists provided verbal consent. This study was conducted from September 2022 to June 2023. Before CT TRUST was initiated, patient perceptions of relational empathy were collected in September and October 2022 as a baseline. The intervention occurred from November 2022 to May 2023. The study took place across 3 diverse hospitals within a single health system: a 1315-bed quaternary-care medical center, a 489-bed community tertiary-care hospital, and a 100-bed community hospital. This study adhered to the Standards for Quality Improvement Reporting Excellence (SQUIRE) reporting guideline.

### Participants

Study participants included hospitalists, English-speaking adult patients admitted to medical floors under the care of hospitalists, and the PCPs of these patients. The study team introduced the project at hospitalist meetings at each hospital, addressing questions and ensuring verbal consent from all day-shift hospitalists practicing on medical floors. Patients on medical floors, excluding those with specific restrictions (eg, police custody), were considered potentially eligible to participate. A research assistant approached patients for study participation. Patients who were unable to consent or lacked a PCP were excluded. Of 662 patients approached, 562 (84.9%) were eligible and 333 (59.3%) consented to participate. Among these, the research assistant contacted their PCPs, and 150 patients’ PCPs (45.0%) agreed to participate (Figure).

**Figure.**
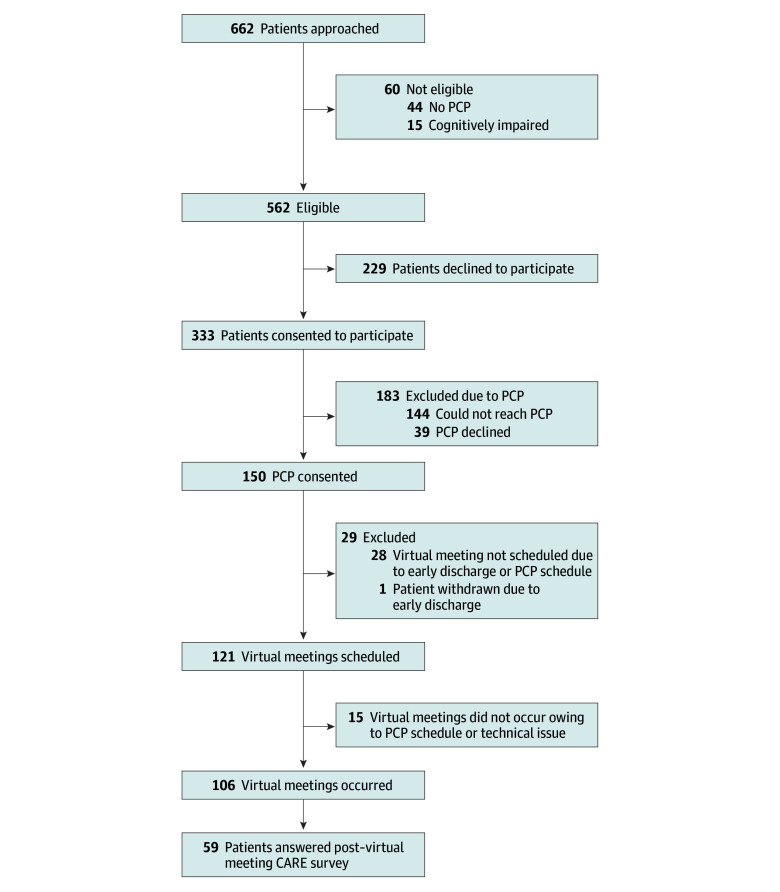
Care Transitions Technology to Reinforce Understanding and Support Trust Patient Enrollment Flowchart CARE indicates Consultation and Relational Empathy; PCP, primary care practitioner.

### Intervention and Implementation

The CT TRUST intervention involved a structured, video-mediated interaction (ie, virtual meeting) among the patient, family caregiver (if available), hospitalist, and PCP, conducted on or before the day of discharge. During this interaction, the hospitalist provided a brief review of the patient’s hospital stay, including the diagnosis, procedures performed, and medication changes. The PCP then asked questions and endorsed the hospitalist’s care. Together, they explained the key components of the postacute care plan to the patient. No formal training of the hospitalists or PCPs was undertaken.

Of 150 patients whose PCPs agreed to participate, virtual meetings were scheduled for 121 patients (81.7%) by the research assistant, and 106 of these patients (87.6%) successfully completed their virtual meetings before discharge. After discharge, 59 patients (55.7%) completed the postdischarge survey (Figure). These 106 patients were cared for by a total of 56 hospitalists and 95 PCPs who participated in the CT TRUST intervention.

### Data Collection and Measures

Patients’ demographic information, including age, sex, gender, race and ethnicity, marital status, and health insurance type, was collected through the electronic health record (EHR) system or self-reporting. EHR data were used to compare demographic differences between patients who consented and those who did not. Additionally, for consented patients, analysis examined whether significant demographic differences existed between those whose PCPs consented vs those whose PCPs did not.

We used the Consultation and Relational Empathy (CARE) measure^19^ to assess patients’ perceptions of relational empathy and human aspects of medical care. It evaluates clinicians’ performance (ie, behavior) as perceived by patients.^4,19^ Developed based on a broad definition of empathy within the context of therapeutic relationships, the measure aims to provide a holistic, patient-centered evaluation that is meaningful to patients irrespective of their social class.^19,20,21^ Its use has been described among PCPs and medical specialists.^22^ The instrument consists of 10 questions, rated by patients on a 5-point Likert scale (1 indicates poor; 5, excellent).

We used the Acceptability of Intervention Measure (AIM), Intervention Appropriateness Measure (IAM), and Feasibility of Intervention Measure (FIM)^23^ to capture clinicians’ perceptions. These implementation measures are commonly used as early indicators of implementation success in pilot studies. The implementation measures were scored with a Likert scale ranging from 1 to 5 (1 indicates strongly disagree; 5, strongly agree). Higher mean scores indicate greater readiness for implementation.

All study instruments were programmed into the REDCap^24^ platform (Vanderbilt), a secure, Health Insurance Portability and Accountability Act–adherent web-based application. Baseline CARE data were collected by the research assistant from a convenience sample at the 3 participating hospitals. Postintervention CARE data were gathered through postdischarge phone calls to participating patients, during which patients’ qualitative comments about the intervention were also recorded. For AIM, IAM, and FIM assessments, a web survey link was distributed to hospitalists and PCPs who participated in virtual meetings with patients at the end of intervention phase. Additionally, feedback from hospitalists and PCPs was collected after each virtual meeting.

### Statistical Analysis

The length of the CT TRUST virtual meeting was recorded, and the mean was calculated. Descriptive statistics were generated for each survey item. Standard analytic approaches from previous literature were applied to the CARE measure^19,20,21^ and AIM, IAM, and FIM responses.^23^ Changes in CARE scores from baseline were analyzed using 2-sample *t* tests. Bivariate analyses assessed associations between clinician specialty and respondents’ perceptions of the intervention. Data analysis was conducted using R software version 4.4.0 (R Project for Statistical Computing), and a 2-sided α <. 05 was considered statistically significant. Data were analyzed from September 2023 to April 2025.

We used conventional content analysis^25,26^ to analyze qualitative comments from patients, family caregivers, hospitalists, and PCPs. Comments were documented verbatim and summarized into common themes that reflected shared experiences and perspectives. Representative quotes were selected to highlight these themes.

## Results

Before conducting CT TRUST, 200 patients agreed to complete the CARE survey. The mean (SD) age was 60.2 (15.6) years. Of these, 112 (57.0%) were female and 86 (43.0%) were male. The racial and ethnic composition was as follows: 1 American Indian or Alaska Native individual (0.5%) , 5 Asian individuals (2.5%), 70 Black individuals (35.0%), 2 Hispanic individuals (1.0%), and 124 White individuals (62.0%). The mean total CARE score was 40.9, with an SD of 9.51 and a 95% CI of 39.5 to 42.2.

The 106 patients who received the CT TRUST intervention had a mean (SD) age of 63.2 (14.9) years. Of these, 51 (48.1%) identified as female and 55 (51.9%) identified as male. There were 37 Black patients (34.9%), no Hispanic patients, and 69 White patients (65.1%). Regarding insurance coverage, 70 patients (66.0%) were insured by Medicare, and 12 patients (11.3%) were covered by Medicaid. Additionally, 56 patients (52.8%) had an established relationship with their PCP for 3 or more years. Detailed participant demographics are presented in Table 1.

**Table 1.** Participant Demographics

Characteristic	Participants, No. (%) (N = 106)
Age, mean (SD), y	63.2 (14.9)
Sex	
Female	51 (48.1)
Male	55 (51.9)
Race	
Black	37 (34.9)
White	69 (65.1)
Hispanic or Latino ethnicity	
Yes	0
No	106 (100)
Marital status	
Divorced	10 (9.4)
Married	48 (45.3)
Single	38 (35.8)
Windowed	6 (5.7)
Other or prefer not to answer	4 (3.8)
Health insurance type	
Commercial	20 (18.9)
Medicaid	12 (11.3)
Medicare	70 (66.0)
Other	1 (0.9)
Unknown	3 (2.8)
Time with PCP, y	
<1	18 (17.0)
1-3	32 (30.2)
3-5	31 (29.2)
>5	25 (23.6)

A comparison of demographics between consented and nonconsented patients revealed that consented patients were younger. Additionally, there was a significant difference in health insurance composition. No significant differences were observed in sex or race and ethnicity between the groups (eTable 1 in Supplement 1). Furthermore, there were no significant demographic differences between patients whose PCPs consented and those whose PCPs did not (eTable 2 in Supplement 1).

Of 106 virtual meetings conducted, 66 (62.3%) were scheduled successfully with 1 attempt, 38 (35.8%) required 2 attempts, and 2 (1.9%) needed 3 attempts. Additionally, 7 meetings had to be rescheduled due to technical issues or PCP availability conflicts. The mean (SD) length of the CT TRUST virtual meetings was 8 (2.8) minutes, with an IQR of 5.8 to 9.0 minutes and a total duration range of 5 to 20 minutes.

Of 106 patients with meetings, 59 (55.7%) completed the CARE Measure postdischarge. Analysis of the postdischarge responses showed a substantial increase in overall CARE scores, with mean (SD) scores increasing from 40.9 (9.5) at baseline to 47.5 (5.3) postintervention, with a difference of 6.6 (95% CI, 4.9-8.3) (*P* < .001) (Table 2).

**Table 2.** Comparison of CARE Measure for CT TRUST Participants vs Baseline Group

Measure	Score, mean (SD)	
	Baseline group (n = 200)	CT TRUST cohort (n = 59)
Making you feel at ease…	4.1 (1.0)	4.8 (0.6)
Letting you tell your story…	4.1 (1.1)	4.9 (0.4)
Really listening…	4.1 (1.1)	4.8 (0.4)
Interested in you as whole person…	4.0 (1.1)	4.7 (0.7)
Fully understanding your concerns…	4.0 (1.1)	4.6 (0.7)
Showing care and compassion…	4.1 (1.1)	4.8 (0.5)
Being positive…	4.2 (1.0)	4.9 (0.4)
Explaining things clearly…	4.1 (1.1)	4.7 (0.8)
Helping you take control…	4.0 (1.1)	4.6 (0.8)
Making a plan of action with you…	4.0 (1.1)	4.7 (0.8)
Total, mean (SD) [95% CI]^a^	40.9 (9.5) [39.5-42.2]	47.5 (5.3) [46.5-48.5]

Abbreviations: CARE, Consultation and Relational Empathy; CT, Care Transitions; TRUST, Technology to Reinforce Understanding and Support Trust.

^a^ The difference was 6.6 (95% CI, 4.9-8.3); 2-sample *t* test: *P* < .001.

The AIM, IAM, and FIM surveys were completed by 50 of 95 PCPs (52.6%) and 42 of 56 hospitalists (75.0%), showing a high response rate from both groups. Overall, ratings were generally positive to moderate regarding intervention acceptability, appropriateness, and feasibility. Table 3 highlights differences in perceptions of the CT TRUST intervention between PCPs and hospitalists. PCPs expressed more favorable views on the intervention’s acceptability (mean [SD] score, 4.1 [0.9] vs 3.6 [0.8]; difference, 0.5 [95% CI, 0.15-0.85]; *P* = .006) and appropriateness (mean [SD] score, 4.1 [0.9] vs 3.7 [0.9]; difference, 0.4 [95% CI, 0.04-0.76]; *P* = .03) compared with hospitalists. Ratings for feasibility were similar between groups.

**Table 3.** Hospitalist and PCP Perspectives to CT TRUST

Measure	PCPs (n = 50)^a^	Hospitalists (n = 42)^a^	Difference (95% CI)	*P* value^b^
Acceptability of Intervention Measure				
Respondents, No.	49	40	NA	NA
Mean (SD) [95% CI] score	4.1 (0.9) [3.9-4.4]	3.6 (0.8) [3.3-3.8]	0.5 (0.2-0.9)	.006
Intervention Appropriateness Measure				
Respondents, No.	47	42	NA	NA
Mean (SD) [95% CI] score	4.1 (0.9) [3.9-4.4]	3.7 (0.9) [3.4-3.9]	0.4 (0.0-0.8)	.03
Feasibility of Intervention Measure				
Respondents, No.	46	42	NA	NA
Mean (SD) [95% CI] score	3.9 (1.0) [3.6-4.2]	3.5 (0.9) [3.2-3.8]	0.4 (0.0-0.8)	.05

Abbreviations: CT, Care Transitions; NA, not applicable; PCP, primary care practitioner; TRUST, Technology to Reinforce Understanding and Support Trust.

^a^ Exclude participant who did not answer all questions in each measure.

^b^ For mean, 2-sample *t* test.

### Qualitative Feedback

Qualitative feedback from patients, PCPs, and hospitalists underscored the positive impact of the CT TRUST intervention on communication, care coordination, and patient confidence. These insights not only reflect the intervention’s immediate benefits but also point to its potential value as a routine component of the discharge process.

#### Make This a Routine Part of the Discharge Process

Both patients and practitioners strongly advocated for integrating the CT TRUST intervention into standard discharge procedures, expressing enthusiasm for its potential to enhance care transitions. For example, a PCP asked “Why is this not routine to begin with!? This is great!” Similarly, a patient said “They should do this after every hospital visit.” A patient who was previously assigned a new PCP said “The thing y’all did was perfect. That was the first time I saw my doctor face to face. I wish more hospitals did this type of thing more often.” Furthermore, a hospitalist said “Thanks for setting this up, I really enjoyed the call and appreciate this project. Steps in the right direction.”

#### Increased PCP Awareness of Patient’s Hospitalization

Participants highlighted how the intervention improved PCP awareness of their patients’ conditions, fostering a sense of reassurance and clarity. One patient said “I haven’t seen Dr. [name redacted] since this all started. I’ve been going from hospital to hospital. I’m glad he finally knows what’s going on” Another patient said “My PCP does not know I am here right now. He needs to be in the loop and know what is going on. It has been hard for me to reach him….” A different patient said “This conversation is what will give me the peace of mind to travel back to [location redacted], knowing that Dr. [name redacted] finally knows what’s been going on with me” And a PCP said “So thrilled this is happening, would love to participate and I actually want to ask the hospital team a few questions.“

#### Enhanced Information Exchange and Collective Action on Care Plans

Participants felt that the intervention facilitated real-time communication and collaborative decision-making, enabling hospitalists and PCPs to cocreate actionable care plans in the presence of patients. For example, one PCP said “I loved this little 5 minute chat, it was extremely helpful to hear your [the hospitalist] perspective on things” A hospitalist said “I feel like I really improved patient’s care by talking with the PCP while the patient watched. We even scheduled the patient’s follow-up appointment right then.” Furthermore, a patient said “This is an amazing program. My PCP is in Illinois and I want to make sure she is on the same page as these doctors. It will keep everything running smoothly.”

#### Patients Felt Cared for and Empowered to Share Concerns

Patients expressed deep appreciation for the personalized attention and the opportunity to voice their concerns during the intervention. As an example, one patient reported “I think this is the most useful thing. All of you have been so great about taking care of me and this reassures me that you are giving me the best care. This shows you care,” and another patient said “...this brings me comfort knowing you guys reaching Dr. [name redacted] and telling him what has been going on. It takes the pressure off me to update him and send him my medical records.” According to a hospitalist, “Interactions with patient and family became much more appreciative after this, and they thanked me for the meeting the next day.”

#### Increased Assurance and Confidence in Care

Patients reported feeling more secure and confident about their care and follow-up steps after participating in the intervention. One patient reported “The call went wonderful, he [their PCP] really paid attention to what the doctor [the hospitalist] said on the call. Up until that point I was scared to go home. Afterwards, I was confident that I was getting the right care.” Another patient said “Seeing the loop of communication in real life makes me confident in the care I receive. This is evidence all members involved in my care are on the same page and up to date.” A patient’s family caregiver said “I am the one in charge of keeping track of things for him [the patient]. This is so helpful. I feel so much better.”

## Discussion

The CT TRUST quality improvement study found notable improvements in patient perceptions of care associated with this video-based intervention, particularly in relational empathy, and highlighted the intervention’s acceptability, appropriateness, and feasibility as viewed by key stakeholders. For context, a 2017 review of 64 independent CARE studies reported a mean physician score of 39.68.^22^ Compared with normative values published by the creators of the CARE Measure,^27^ preintervention scores ranked in the 10th percentile, while postintervention scores reached the 75th percentile. This increase in CARE survey scores indicates a significant positive association of the intervention with patients’ perceptions of relational empathy and the human aspects of care. Operational data further supported the intervention’s feasibility. Most virtual meetings were scheduled with a single attempt, and only a small fraction required rescheduling due to technical issues or scheduling conflicts. The median meeting length was 8 minutes, suggesting the intervention’s time demands are manageable within clinical workflows. The findings underscored the potential of virtual care coordination to streamline care transitions through enhanced communication and collaboration between hospitalists and PCPs. Both quantitative and qualitative findings highlighted the value of virtual meetings in addressing gaps in information exchange, increasing patient confidence, and improving clinician perceptions of care coordination. These results support the feasibility and potential of incorporating such interventions as a routine component of discharge procedures.

A key strength of CT TRUST was its ability to bridge communication gaps between hospitalists and PCPs. The virtual meetings facilitated real-time information sharing, ensuring that PCPs were better informed about patients’ hospitalizations, treatments, and follow-up needs. This alignment between inpatient and outpatient practitioners is critical for maintaining continuity of care, particularly for patients with complex medical conditions. Feedback from both clinicians and patients highlighted the intervention’s role in creating actionable, collaborative care plans, further demonstrating its value as a mechanism for improving discharge processes. The intervention also emphasized collective action, with hospitalists and PCPs working together to address patient needs. For example, patients initially hesitant about necessary follow-up procedures, like colonoscopies, voiced greater understanding and planned adherence after the joint discussion between the hospitalist and PCP. This aligns with findings from other studies suggesting that effective communication between hospital-based and outpatient practitioners can reduce readmissions and improve patient outcomes.^4,11,12,13,14,16^

One of the most compelling aspects of the CT TRUST intervention was its association with patient trust and confidence. By incorporating relational elements into the discharge process, the intervention helped patients feel more cared for and reassured about their follow-up care. Many patients expressed gratitude for the opportunity to engage simultaneously with both their hospitalist and PCP, noting that this interaction alleviated their anxiety and bolstered their confidence in the care they were receiving. This is important, given the challenges many patients face in coordinating care after discharge,^4^ particularly when dealing with complex medical conditions or having limited access to health care resources. The intervention’s focus on relational care extended beyond information sharing to include active efforts to build rapport, listen to patient concerns, and demonstrate empathy. Patients felt heard and valued, which enhanced their confidence and their perception of the health care system. This approach fostered trust, which is foundational to effective care transitions and improved health outcomes.^28,29,30^

Both hospitalists and PCPs expressed positive perceptions of CT TRUST, with PCPs particularly noting its acceptability and appropriateness. This is a promising outcome, as clinician buy-in is essential for the long-term sustainability and integration of such interventions into routine practice. The intervention potentially can save PCP’s time by mitigating the frequency of inquiries from patients and family caregivers related to posthospital care. While hospitalists also expressed favorable perceptions, they rated the intervention slightly lower than PCPs. This could reflect the added burden of coordinating these virtual meetings within their already demanding schedules. These differences may reflect the varying roles and priorities of these practitioners in transitional care, emphasizing the need for tailored strategies to ensure buy-in from both groups.

From a policy standpoint, the results of CT TRUST suggest a need to formally incorporate structured communication processes into existing care transition frameworks. Specifically, the Centers for Medicare & Medicaid Services could consider integrating a direct interaction component with hospitalists, patients, and PCPs into the requirements for Transitional Care Management billing. Notably, some PCPs declined to participate because of the lack of reimbursement; Transitional Care Management must occur after hospital discharge. One option would be for the CT TRUST intervention to count as a Transitional Care Management visit. By incentivizing these interactions, the Centers for Medicare & Medicaid Services encourages broader adoption of such practices, ultimately improving care continuity, and fostering stronger patient-clinician relationships. This policy shift could serve as a catalyst for embedding relational, patient-centered care into standard discharge protocols across health care systems.

### Limitations

While the CT TRUST intervention showed feasibility and positive outcomes, some limitations must be considered. First, the study relied on a convenience sample of patients and practitioners, which may limit the generalizability of the findings. Moreover, it only included patients who have established PCP relationships, which limits the applicability of this intervention. Additionally, the relatively small sample size of patients (106) and practitioners (95 PCPs, 56 hospitalists) may not fully capture the broader dissemination feasibility. Future studies with larger and more diverse populations could provide further insights into its effectiveness across different demographics and health care settings. Moreover, although it was successful in improving communication and patient confidence, the effects on clinical outcomes, such as readmissions or emergency department visits, were not measured. Future research should explore these outcomes to determine whether CT TRUST can lead to sustained improvements in health care utilization and patient outcomes. Furthermore, while qualitative feedback indicated strong support for making CT TRUST a routine part of discharge processes, the logistical challenges may impede scaling such an intervention to a larger patient population or multiple hospitals. Further evaluation of the cost-effectiveness and logistical feasibility of scaling-up is needed to understand its broader applicability.

## Conclusions

This quality improvement study found that the CT TRUST intervention was associated with enhanced communication between hospitalists and PCPs, fostering patient trust, emphasizing relational aspects of health care, and contributing to improved perception of care transitions. By addressing both the technical and human dimensions of care coordination, CT TRUST demonstrated its potential to improve patient outcomes and satisfaction. With further refinement, policy support, and broader implementation, it could serve as a model for integrating patient-centered, relational approaches into routine discharge processes, ultimately advancing the quality and continuity of care.

## Notes

Supplement 1. **eTable 1.** Demographics of Consented vs Nonconsented Patients **eTable 2.** Demographics of Consented Patients Whose PCPs Consented vs Those Whose PCPs Did Not Consent

Supplement 2.
Data Sharing Statement
