# Supplementary material for: Virtual Patient-PCP-Hospitalist Care Transition Meeting Before Hospital Discharge
Source: JAMA Netw Open. 2025 Jun 13;8(6):e2515848. doi: 10.1001/jamanetworkopen.2025.15848 (PMC12166488; doi:10.1001/jamanetworkopen.2025.15848)
Supplement: Supplement 1. — eTable 1. Demographics of Consented vs Nonconsented Patients eTable 2. Demographics of Consented Patients Whose PCPs Consented vs Those Whose PCPs Did Not Consent [file jamanetwopen-e2515848-s001.pdf]

Supplemental Online Content

Li J, Reuter MD, Schmidt JM, et al. Virtual patient-PCP-hospitalist care transition meeting before hospital discharge. *JAMA Netw Open*. 2025;8(6):e2515848. doi:10.1001/jamanetworkopen.2025.15848

eTable 1. Demographics of Consented vs Nonconsented Patients
eTable 2. Demographics of Consented Patients Whose PCPs Consented vs Those Whose PCPs Did Not Consent

This supplemental material has been provided by the authors to give readers additional information about their work.

© 2025 Li J et al. *JAMA Network Open*.

eTable 1. Demographics of Consented vs. Non-Consented Patients

	Patient Consented (333)+	Patient Not Consented (230)	P value*
**Age – mean (SD)**	62.0 (14.89)	66.0 (14.67)	**0.002**
**Sex**			0.23
Female (N, %)	187 (56.3)	117 (50.9)	
Male (N, %)	145 (43.7)	113 (49.1)	
**Gender**			0.40
Man (N, %)	146 (44.0)	112 (48.7)	
Woman (N, %)	185 (55.7)	118 (51.3)	
Agender (N, %)	1 (0.3)		
**Race**			0.21
Black (N, %)	128 (38.6)	74 (32.2)	
White (N, %)	203 (61.1)	151 (65.7)	
Other (N, %)	1 (0.3)	5 (2.1)	
**Ethnicity**			0.38
Hispanic (N, %)	1 (0.3)	3 (1.3)	
Non-Hispanic (N, %)	331 (99.7)	227 (98.7)	
**Health insurance**			**0.004**
Commercial (N, %)	72 (21.6)	61 (26.6)	
Medicaid (N, %)	41 (12.3)	27 (11.8)	
Medicare (N, %)	207 (62.2)	118 (51.5)	
Other (N, %)	8 (2.4)	5 (2.2)	
Unknown (N, %)	5 (1.5)	18 (7.9)	

+ One patient withdrew consent due to early discharge

* For mean, Analysis of Variance (ANOVA) test; For proportion, Chi-square test

© 2025 Li J et al. *JAMA Network Open*.

eTable 2. Demographics of Consented Patients Whose PCPs Consented vs. Those Whose PCPs Did Not Consent

	Patient Consented, PCP Consent (149)	Patient Consented, PCP Not Consented (183)	P value*
**Age – average (SD)**	63.4 (14.68)	61.5 (15.01)	0.2490
**Sex**			0.0610
Female (N, %)	75 (50.3)	112 (61.2)	
Male (N, %)	74 (49.7)	71 (38.8)	
**Gender**			0.0788
Man (N, %)	75 (50.3)	71 (38.8)	
Woman (N, %)	74 (49.7)	111 (60.7)	
Agender (N, %)	0	1 (0.5)	
**Race**			0.2948
Black (N, %)	52 (34.9)	76 (41.5)	
White (N, %)	97 (65.1)	106 (57.9)	
Asian (N, %)	0	1 (0.5)	
**Ethnicity**			1
Hispanic (N, %)	0	1 (0.5)	
Non-Hispanic (N, %)	149 (100)	182 (99.5)	
**Marital Status**			0.5425
Divorced (N, %)	16 (10.7)	22 (12.0)	
Married (N, %)	67 (45.0)	74 (40.4)	
Single (N, %)	52 (34.9)	64 (35.0)	
Widowed (N, %)	10 (6.7)	21 (11.5)	
Other or Prefer not to answer (N, %)	4 (2.7)	2 (1.1)	
**Health insurance type**			0.0998
Commercial (N, %)	31 (20.8)	41 (22.4)	
Medicaid (N, %)	14 (9.4)	27 (14.8)	
Medicare (N, %)	99 (66.4)	107 (58.5)	
Other (N, %)	2 (1.4)	6 (3.3)	
Not on file (N, %)	3 (2.0)	2 (1.1)	
**Length with PCP**			0.2551
< 1 year (N, %)	26 (17.4)	33 (18.0)	
1 – 3 years (N, %)	44 (29.5)	44 (24.0)	
3 – 5 years (N, %)	44 (29.5)	34 (18.6)	
5 + years (N, %)	35 (23.6)	49 (26.8)	
No answer (N, %)	0	23 (12.6)	

* For mean, Analysis of Variance (ANOVA) test; For proportion, Chi-square test

© 2025 Li J et al. *JAMA Network Open*.
