# Supplementary material for: Virtual Patient-PCP-Hospitalist Care Transition Meeting Before Hospital Discharge
Source: JAMA Netw Open. 2025 Jun 13;8(6):e2515848. doi: 10.1001/jamanetworkopen.2025.15848 (PMC12166488; doi:10.1001/jamanetworkopen.2025.15848)
Supplement: Supplement 2. — Data Sharing Statement [file jamanetwopen-e2515848-s002.pdf]

Data Sharing Statement

Li. Virtual Patient-PCP-Hospitalist Care Transition Meeting Before Hospital Discharge. *JAMA Netw Open*. Published June 13, 2025. doi:10.1001/jamanetworkopen.2025.15848

Data
Data available: Yes
Data types: Deidentified participant data
How to access data: jingli2@uabmc.edu
When available: With publication

Supporting Documents
Document types: None

Additional Information
Who can access the data: anyone requesting data
Types of analyses: for any purpose
Mechanisms of data availability: with approval of proposal
